# Photonic plasmid stability of transformed *Salmonella Typhimurium*: A comparison of three unique plasmids

**DOI:** 10.1186/1471-2180-9-152

**Published:** 2009-07-27

**Authors:** Keesla Moulton, Peter Ryan, Donald Lay, Scott Willard

**Affiliations:** 1 Department of Animal and Dairy Science, Mississippi State University, Box 9815, Mississippi State, MS 39762, USA; 2 USDA-ARS, Livestock Behavior Research Unit, 125 S. Russell St. West Lafayette, IN, 47907 USA

## Abstract

**Background:**

Acquiring a highly stable photonic plasmid in transformed *Salmonella Typhimurium *for use in biophotonic studies of bacterial tracking *in vivo *is critical to experimental paradigm development. The objective of this study was to determine stability of transformed *Salmonella Typhimurium *(*S. typh*-lux) using three different plasmids and characterize their respective photonic properties.

**Results:**

In presence of ampicillin (AMP), *S. typh*-lux with pCGLS-1, pAK1-lux and pXEN-1 plasmids exhibited 100% photon-emitting colonies over a 10-d study period. Photon emitters of *S. typh*-lux with pCGLS-1, pAK1-lux and pXEN-1 without AMP selection decreased over time (P < 0.05), representing only 11 ± 1%, 35 ± 1% and 43 ± 1%, respectively, of original photon emitting properties of the bacterial population by d 10. Photonic emissions were positively correlated with bacterial concentration (P < 0.05) for pAK1-lux, pCGLS-1 and pXEN-1 (r = 0.96, 0.98 and 0.82, respectively). When stratified by high, medium and low density bacteria concentrations, photonic emissions for high density populations containing pAK1-lux, pCGLS-1 and pXEN-1 resulted in differences of photonic emissions across a range of bacterial concentrations (1 × 10^7 ^to 1 × 10^9 ^CFU, P < 0.05) with positive correlations (P < 0.05) of (r = 0.72, 0.46 and 0.72, respectively). The correlation of photonic emissions with bacterial concentrations for samples with medium and low density bacteria (pAK1-lux, pCGLS-1, and pXEN-1 plasmids) imaged in tubes were also positively correlated (medium; r = 0.69, 0.49, 0.46, low; r = 0.90, 0.71, 0.68, respectively; P > 0.05); although photonic emissions across a range of bacterial concentrations were not different (1 × 10^4 ^to 1 × 10^6 ^CFU, P > 0.05). For very low density bacterial concentrations imaged in 96 well plates photonic emissions were positively correlated with bacterial concentration (P < 0.05) for pAK1-lux, pCGLS-1, and pXEN-1 plasmids (r = 0.99, 0.99, and 0.96, respectively), and photonic emissions across a range of bacterial concentrations (1 × 10^3 ^to 1 × 10^5 ^CFU) low to high were different in the 96-well plate format (P < 0.05).

**Conclusion:**

These data characterize photon stability properties for *S. typh*-lux transformed with three different photon generating plasmids that may facilitate real-time *Salmonella *tracking using *in vivo *or *in situ *biophotonic paradigms.

## Background

Researchers are increasingly interested in observing biological processes in real-time. The development of reporter systems such as fluorescence and bioluminescence allows for imaging and measuring of various biological activities. Various reporter plasmids developed from *Photorhabdus luminescens *and plasmids developed from firefly luciferases are being utilized for *in vitro *and *in vivo *bioluminescence research paradigms [1-3] yet comparisons of plasmid functional characteristics and stability post-transformation are not always characterized or compared.

The biochemical basis of the bacterial-bioluminescence system has been characterized and is well documented [4]. Along with the lux genes, antimicrobial genes are added to the gene cassettes for plasmid preparation in bacteria to provide selective pressure (e.g. ampicillin resistance) for procedural manipulations. However, stability of recombinant plasmids is sometimes an issue, as pMG36e-lux-AB was found to be very stable in *Lactococcus lactis *MG1363, while pMG36e-luxCDABE was unstable in the absence of selective pressure in the form of antibiotic [5] and therefore may not be a suitable plasmid for work where the short term absence of antibiotic selective pressure may not be desired or is not possible (e.g., *in vivo *imaging).

The plasmid pCGLS-1 is an insert of approximately 11 kb of *X. luminescens *DNA and ampicillin is used for selection The genes for production of light encode the two subunits of luciferase and the enzymes of the fatty acid reductase complex [6]. The pAK1-lux plasmid was developed as a broad host range plasmid by using a pBBR1 replicon to constitutively and inducibly express gfpmut3a and luxCDABE genes from the Plac promoter [7] for gram negative bacterium, and ampicillin is used for selection. Plasmid pXEN-1 is a shuttle plasmid carrying the modified *luxABCDE *operon for engineering bioluminescent gram positive bacteria. The original gene sequence of gram negative *P. luminescens *lux *CDABE *genes are modified to AGGAGG that can be optimally recognized in *g*ram positives. There is no apparent terminator for the *luxABCDE *operon on the plasmid and ampicillin is used for selection in *E. coli *while chloramphenicol is used for selection of the autonomous replicating plasmid in gram positives [8].

The objective of this study was to determine the stability of transformed *Salmonella Typhimurium *(*S. typh*-lux) using three different plasmids (pAK1-lux, pXEN-1, and pCGLS-1) in the presence and absence of selective pressure *in vitro*. In addition, we sought to determine the respective photonic properties (luminescent:bacterial concentration correlations and minimum and maximum luminescent thresholds) of each plasmid using different imaging platforms (e.g. 1.5 ml black microcentrifuge tubes vs 96 well plates, etc.) and by varying concentrations of *S. typh*-lux bacteria.

## Methods

### Transformation and Selection of *Salmonella Typhimurium*

*Salmonella Typhimurium *(ATCC # 14028; Manassas, VA) were transformed with plasmid pAK1-lux(4), pXEN-1 (Xenogen Bioware™), and pCGLS-1 [6] using an electroporation protocol described in detail previously [9,10]. Following transformation, the bacteria were spread on Brilliant Green Agar (BG) + Ampicillin Sodium Salt, (AMP; 2 μg/ml; Sigma-Aldrich, Inc. St. Louis, MO) for selection. From the plate both AMP and no AMP Luria Bertani (LB) broth cultures were inoculated to be used in the stability experiment (Experiment 1) and AMP LB broth cultures were used for photonic and bacterial concentration correlations in black microcentrifuge tubes and black 96-well plates (Experiment 2).

### Experiment 1: Inoculum, imaging, plating and counting procedure for plasmid stability

One colony (*S. typh*-lux) was transferred to 20 ml of LB broth and LB + AMP and shaken in an orbital shaker at 37°C for 24 h. From each 24-h inoculum, 2 repetitions of 8 wells filled with 100 μl in respective columns of a black 96-well plate were used for imaging, and 7 wells per plate (n = 2) were used for subsequent serial dilution and plating for bacterial counts (n = 14). One well was transferred back to an inoculum tube to sub-culture for another 24 h up to 10 d. All other wells were filled with 100 μl of sterile broth. The 96-well plates were then imaged using a XR/MEGA-10Zero™ (Stanford Photonics, Inc, Palo Alto, CA) photonic imaging system at 1 × 1 binning and an acquisition time of 5 sec. Each well was serially diluted in 900 μl of LB or LB+AMP broth. Three-dilutions were spread on BG or BG+AMP agar and incubated at 37°C overnight. The incubation tubes were placed in a 37°C orbital shaker and the imaging, serial dilution, and plating was conducted every 24 h up to 10 d. On each day following plating, the agar plate colonies were counted, imaged, the number of emitting colonies recorded and bacterial concentrations calculated. The photonic images of the black 96-well plates were analyzed using Image J software (NIH) and reported as relative light units per sec (RLU/s), the emissions from the comparison blank sterile broth wells (i.e., background) were subtracted from the bacterial emitting wells to correct for background photonic emissions. Percent emissions were calculated daily as: (number of emitting colonies/total number of colonies)*100. These procedures were carried out for each of the three plasmids analyzed.

### Experiment 2: Inoculum, imaging, plating and counting procedure for plasmid characterization

One colony (*S. typh*-lux) was transferred to 20 ml of LB + AMP and shaken in an orbital shaker at 37°C for 24 h. From this inoculum, 6 separate sets were serially diluted (n = 15) as high, medium, and low density bacterial populations in LB+AMP broth (1-ml black microcentrifuge tubes) and prepared for imaging. Another very low density set (with 4 serial dilutions) of 100 μl per well (n = 15) were transferred to black 96-well plates for further comparisons of the lower-limits of photonic detection relative to bacterial concentration. The tube sets, including 5 tubes with sterile broth for background correction, were then imaged using a XR/MEGA-10Zero™ (Stanford Photonics, Inc, Palo Alto, CA) imaging system at 1 × 1 binning and an acquisition time of 2 to 30 s. The 96-well plates were imaged under the same parameters, however a 30 s acquisition time was utilized with these low concentration/low light detection determinations. From each tube or well, 100 μl was serially diluted in 900 μl of LB or LB+AMP broth. Three-dilutions were then plated on BG or BG+AMP agar and incubated at 37°C overnight. The following day, the agar plate colonies were counted, imaged, the number of emitting colonies recorded, and bacterial concentrations calculated. The photonic images of the black micocentrifuge tubes and 96-well plates were analyzed using Image J software (NIH) and reported in RLU/s. The emissions from the comparison blank sterile broth tubes and wells (i.e. background) were subtracted from the bacterial emitting tubes to correct for background photonic emissions. These procedures were carried out for each of the three plasmids analyzed.

### Statistical Analysis

Percent photonic emissions, well photonic emissions, and bacterial concentrations were analyzed over time by repeated measures ANOVA using the mixed procedure. Pearson Correlations were used to determine coefficients for emitting *S. typh*-lux bacterial concentrations and well photonic emissions (SAS 9.1, Cary, NC). Tube photonic emissions and bacterial concentrations in tubes were analyzed by Mixed Procedure with Least Square Means to determine differences. Pearson Correlations were used to determine coefficients for emitting *S. typh*-lux bacterial concentrations and tube or well photonic emissions (SAS 9.1, Cary, NC).

## Results and discussion

**Figure 1:**
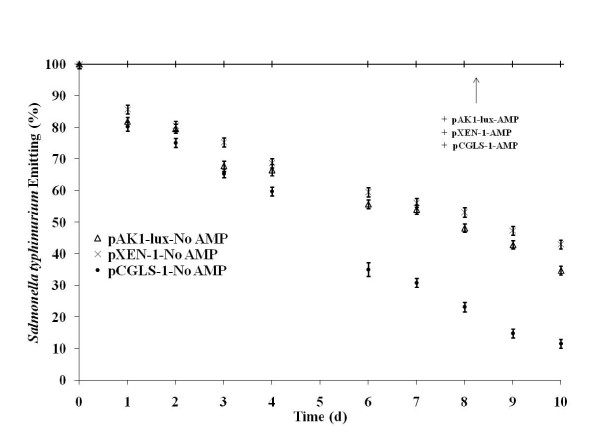
**Percentage of bacteria emitting photons**. Percentage of photon-emitting *Salmonella typhimurium *and lux-plasmid (pAK1-lux, pXEN-1, or pCGLS-1) following imaging in the presence of ampicillin and without ampicillin selection for 10 consecutive days *in vitro *(P < 0.05).

**Figure 2:**
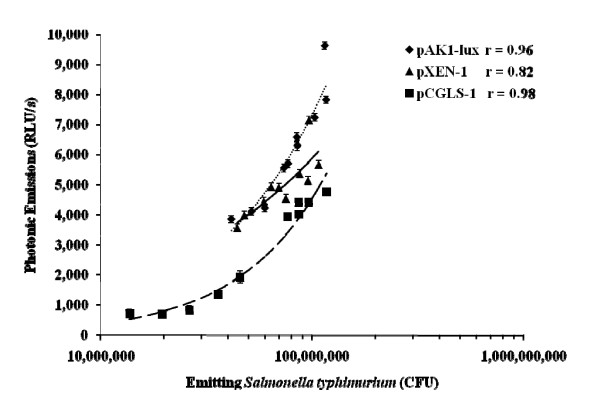
**Correlation between luminescence and bacterial numbers**. The correlation of photon-emitting *Salmonella typhimurium *and lux plasmid (pAK1-lux, pXEN-1, or pCGLS-1) following imaging without ampicillin selection in wells of 96-well plate (P < 0.05).

Previous research from our laboratory concerning the stability of pAK1-lux plasmid in *E. coli *over a continual sub-culture without antibiotic selective pressure indicated a continual gradual decline in the percent of bacterial emissions from 100% to 66% by d 8 [10]. Moreover, *Salmonella Typhimurium *with plasmid pCGLS-1 and pAK1-lux were similarly evaluated for stability and indicated a decline in percent of photonic emissions by day 6 of 39 and 55.5%, respectively [11]. Our current results are similar with a continual decline in percent of emissions for all plasmids, however by day 6 the plasmid pCGLS-1 percent emissions were lower than pAK1-lux or pXEN-1, and much lower by day 10 (Table 1 and Figure 1). Moreover, a decline in photonic emissions as well as a decrease in bacterial concentration from d 0 to 10 in Experiment 1 (Table 2) resulted in good correlations between bacterial numbers and photonic emissions (Figure 2). Another bacterium, *Edwardsiella ictaluri *has been imaged *in vitro *and similarly evaluated with the pAK1-lux plasmid resulting with a decline in bioluminescence after 10 days of sub-culturing without antibiotic selective pressure and appears to have a half-life of 18 days [7]. Several *Salmonella *strains were also similarly evaluated without antibiotic selective pressure with the pAK1-lux plasmid and results also demonstrated a continued linear decline of bioluminescence with a half-life estimation of 7 days [12].

**Table 1:** Stability of luminescent bacteria evaluated as percent emitting bacteria.

	Luminescent *Salmonella typhimurium*		
	Day of Culture (% of Total Colonies)		
Plasmid Type	0	6	10
pAK1-lux			
% Emitters	100	56 ± 1^a,x^	35 ± 1^b,x^
% Non-Emitters	0	44 ± 1	65 ± 1
pXEN-1			
% Emitters	100	60 ± 1^a,y^	43 ± 1^b,y^
% Non-Emitters	0	40 ± 1	57 ± 1
pCGLS-1			
% Emitters	100	35 ± 2^a,z^	11 ± 1^b,z^
% Non-Emitters	0	65 ± 2	89 ± 1

Superscripts differ within plasmid ^a,b ^(P < 0.05).

Superscripts differ between plasmids ^x,y,z ^(P < 0.05).

Stability of luminescent *Salmonella typhimurium *with three different plasmids (pAK1-lux, pXEN-1, and pCGLS-1); Percent emitting and non-emitting evaluations at day 0, 6, and 10 of *in vitro *culturing without ampicillin selection.

**Table 2:** Stability of luminescent bacteria evaluated as emitting concentration and luminescence detection.

	Luminescent *Salmonella typhimurium*		
	Day of Culture (Emitting Concentration; CFU)		
Plasmid Type	0	6	10

pAK1-lux	1.2 × 10^8 ^± 7.2 ×10^6a,x^	7.4 × 10^7 ^± 1.1 × 10^7b,x^	4.2 × 10^7 ^± 7.2 × 10^6c,x^
pXEN-1	9.7 × 10^7 ^± 7.2 × 10^6a,x^	7.0 × 10^7 ^± 7.8 × 10^6b,x^	4.4 × 10^7 ^± 7.2 × 10^6c,x^
pCGLS-1	1.2 × 10^8 ^± 7.2 × 10^6a,x^	4.6 × 10^7 ^± 1.1 × 10^7b,y^	1.3 × 10^7 ^± 7.2 × 10^6c,y^

	Luminescent *Salmonella typhimurium*		
	Day of Culture (Photonic Detection; RLU/s)		

Plasmid Type	0	6	10

pAK1-lux	7811 ± 159^a,x^	5550 ± 159^b,x^	3839 ± 158^c,x^
pXEN-1	7149 ± 159^a,y^	4898 ± 171^b,y^	3552 ± 159^c,y^
pCGLS-1	4753 ± 159^a,z^	1921 ± 242^b,z^	708 ± 159^c,z^

Superscripts differ within plasmid ^a,b,c ^(P < 0.05).

Superscripts differ between plasmids ^x,y,z ^(P < 0.05).

Stability of luminescent *Salmonella typhimurium *with three different plasmids (pAK1-lux, pXEN-1, and pCGLS-1) in 96-well format (100 μl/well); Percent emitting and non-emitting evaluations at day 0, 6, and 10 of *in vitro *culturing without ampicillin selection.

A separate study has also evaluated the luminescence signal in broth using the pCGLS-1 plasmid in *Pseudomonas aeruginosa *at various densities through measurements from a luminometer. The detection of the luminescence signal was linearly proportional to bacterial colony forming units [8], and agrees with the results for Experiment 2 in the present study with high and low bacterial densities in broth culture with all three plasmids (pCGLS-1, pXEN-1 and pAK1-lux) in both 1 ml black centrifuge tube or black 96-well plate formats (Table 3). Other scientists using a luminescence assay, via a luminometer plate reader, determined sensitivity as a 3-log reduction in viability whereas the colony-forming unit assay can measure a 6-log reduction in viability [8]. It also appeared that a cytotoxic insult to bacteria causes a loss of viability more readily than it caused loss of luminescence. The decrease in luminescence may be due to exhaustion of ATP supplies from the bacteria (needed for the luciferase enzyme to make luminescence), which cannot be replenished if the cells are fatally damaged [8].

**Table 3:** Detection limits of luminescent *Salmonella typhimurium*.

Item	Bacterial concentration (CFU)	Photonic emissions (RLU/s)
Black tube format (1ml) upper limit (2 s acquisition time)		
Background (used for subtraction of sample)	-	39
pAK1-lux	1.1 × 10^8 ^± 1.0 × 10^7^	7,470 ± 136
pCGLS-1	6.2 × 10^7 ^± 1.2 × 10^7^	6,168 ± 167
pXEN-1	1.0 × 10^8 ^± 1.0 × 10^7^	7,016 ± 136
Black tube format (1. 0 ml) lower limit (2 s acquisition time)		
Background (used for subtraction of sample)	-	33
pAK1-lux	2.7 × 10^6 ^± 1.0 × 10^7^	278 ± 136
pCGLS-1	1.8 × 10^6 ^± 1.0 × 10^7^	327 ± 136
pXEN-1	5.1 × 10^6 ^± 1.0 × 10^7^	148 ± 141

Item	Bacterial concentration (CFU)	Photonic emissions (RLU/s)

96-well black plate format (100 μl) lower limit (30 s acquisition time)		
Background (used for subtraction of sample)	-	6
pAK1-lux	3.8 × 10^3 ^± 2.8 × 10^3^	2.0 ± 1.3
pCGLS-1	2.9 × 10^3 ^± 2.8 × 10^3^	1.1 ± 1.3
pXEN-1	2.8 × 10^3 ^± 2.7 × 10^3^	1.1 ± 1.2

Luminescent *Salmonella typhimurium *with three different plasmids (pAK1-lux, pXEN-1, and pCGLS-1); upper and lower detection limits for black tube format (2 s acquisition time) and low detection limits for black 96-well plate format (30 s acquisition time). The results below are bacterial concentration means ± standard error of the mean and photonic emissions means ± standard error of the mean.

**Figure 3:**
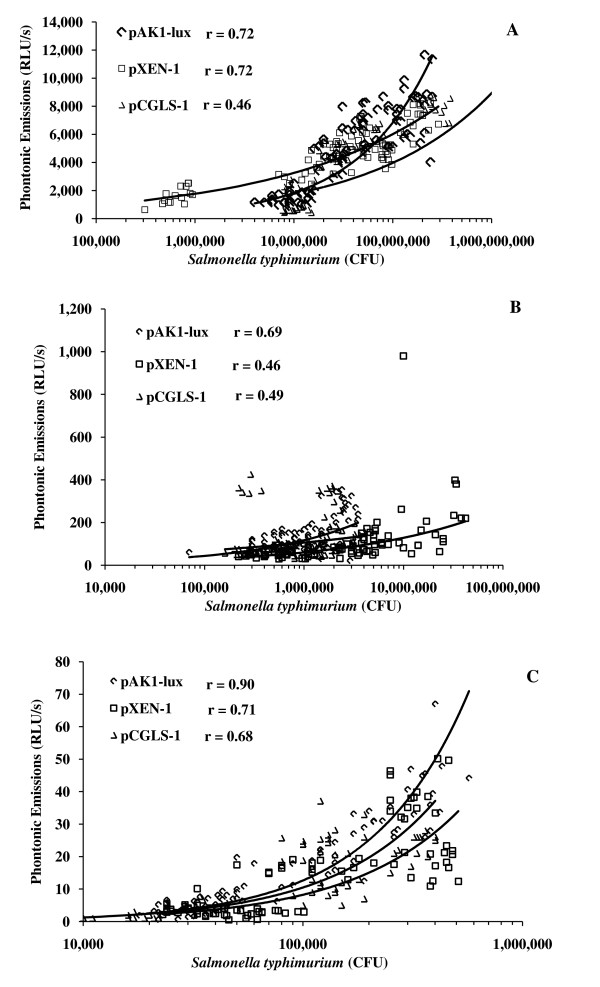
**Correlation between luminescence and bacterial numbers at various densities in black microcentrifuge tubes**. Correlation of photon-emitting *Salmonella typhimurium *and lux plasmid (pAK1-lux, pXEN-1, or pCGLS-1) following imaging of 1 ml aliquots in black microcentrifuge tubes (Panel A) high density (P > 0.05), (Panel B) medium density (P < 0.05), (Panel C) low density of bacteria (P > 0.05).

**Figure 4:**
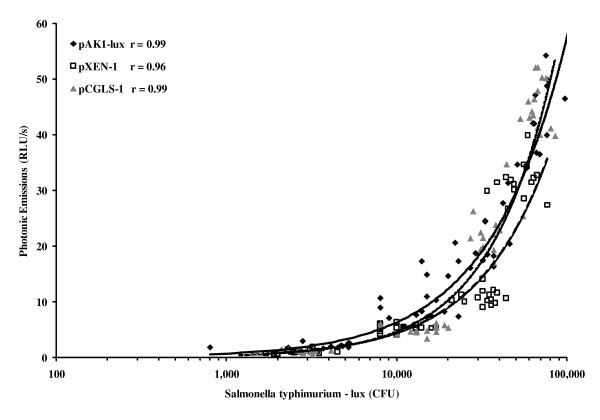
**Correlation between luminescence and bacterial numbers at a very low density in black 96-well plate**. Correlation of photon-emitting *Salmonella Typhimurium *and lux plasmid (pAK1-lux, pXEN-1, or pCGLS-1) following imaging of 100 μl aliquots in wells of black 96-well plate (P < 0.05).

When pAK1-lux was used in *Edwardsiella ictaluri *through 5 orders of magnitude, the relationship of bacterial density and bioluminescence was a linear correlation (r = 0.99) with a minimum detectable number of bacteria in a 96-well plate format of 2500 CFU/ml [7]. Bacteria numbers and bioluminescence correlations were very good (r = 0.99) in 12 strains of *Salmonella *transferred with the pAK1-lux plasmid and for a majority of the strains the minimum detectable bacterial numbers was less than 1500 CFU/ml [12]. The above studies were similar to Experiment 2 results of good correlations for pAK1-lux and pXEN-1 evaluated in the 1 ml black centrifuge tube format as well as the black 96-well plate format (Figure 3 and 4). However the plasmid pCGLS-1 did not have as good a correlation as in the above experiments or relative to the other plasmids in our study for the 1 ml black tube format (Figure 3). We also noted that the minimum detectable concentration for the 1 ml black centrifuge tube is much higher, whereas the minimum detectable concentration for the black 96-well plate format is similar to the above referenced studies [7,8] (Table 3). Other scientists using ten-fold dilutions of a mid-log-phase culture of *Escherichia coli *(pCGLS-1) assayed for bioluminescence using a conventional microtiter luminometer and an ICCD camera obtained similar bioluminescence curves for each system [13]. The dynamic range of the ICCD camera was between approximately 2.6 and 6 log units. The bioluminescence curves were found to closely correlate with viable cell counts, yielding correlation coefficients of 0.98 for both the luminometer and ICCD, respectively, and is similar to results from Experiment 2 in the present study (Figure 3 and 4). The sensitivity of the ICCD camera system was also found to be higher than that of the luminometer, detecting a lower limit of approximately 400 cells with a 1-min signal accumulation time as compared to 104 cells shown with the luminometer [13]. From Experiment 2, the detection of the lower limit of bacterial cells is higher using a 30 s accumulation of signal than the above studies that used a 1 min accumulation signal (Table 3) which is likely the direct result of camera sensitivity. Other scientists have evaluated the minimum number of *S. aureus *RN4220 pXen-1 detectable using a photon-counting ICCD camera. Approximately 400 CFU were detected in the black 96-well plate format. However, using a more sensitive liquid nitrogen-cooled integrating CCD camera (IVIS Imaging system), detection was as few as 80 CFU (5) which is different from the results of Experiment 2 when detecting very low concentrations in the 96-well format of approximately 1,000 CFU (Table 3).

## Conclusion

These data characterize the photon stability properties for *Salmonella Typhimurium *transformed with three different photon generating plasmids. *Salmonella Typhimurium *that is transformed with pAK1-lux and pXEN-1 bioluminescent plasmids are more stable and have better correlations with actual bacterial concentration than the pCGLS-1 plasmid. However for short-term evaluations of 1 to 6 days, all three plasmids may permit real-time *Salmonella *tracking using *in vivo *or *in situ *biophotonic paradigms where antibiotic selective pressure to maintain plasmid incorporation may not be feasible.

## Authors' contributions

KM conceived the study and participated in the design of the study. KM carried out the bacterial-plasmid transformation, participated in the imaging, bacterial serial dilution, plating, counting statistical analysis, data interpretation and drafted the manuscript. SW participated in the design of the study and assisted in statistical analysis as well as helped to draft the manuscript. DL and PR participated in interpretation of data and helped to draft and critically revise the manuscript. All authors read and approved the final manuscript.
